# GeneNet Toolbox for MATLAB: a flexible platform for the analysis of gene connectivity in biological networks

**DOI:** 10.1093/bioinformatics/btu669

**Published:** 2014-10-14

**Authors:** Avigail Taylor, Julia Steinberg, Tallulah S. Andrews, Caleb Webber

**Affiliations:** ^1^MRC Functional Genomics Unit, Department of Physiology, Anatomy and Genetics, University of Oxford, Oxford OX1 3QX, UK and ^2^The Wellcome Trust Centre for Human Genetics, University of Oxford, Oxford OX3 7BN, UK

## Abstract

**Summary**: We present GeneNet Toolbox for MATLAB (also available as a set of standalone applications for Linux). The toolbox, available as command-line or with a graphical user interface, enables biologists to assess connectivity among a set of genes of interest (‘seed-genes’) within a biological network of their choosing. Two methods are implemented for calculating the significance of connectivity among seed-genes: ‘seed randomization’ and ‘network permutation’. Options include restricting analyses to a specified subnetwork of the primary biological network, and calculating connectivity from the seed-genes to a second set of interesting genes. Pre-analysis tools help the user choose the best connectivity-analysis algorithm for their network. The toolbox also enables visualization of the connections among seed-genes. GeneNet Toolbox functions execute in reasonable time for very large networks (∼10 million edges) on a desktop computer.

**Availability and implementation**: GeneNet Toolbox is open source and freely available from http://avigailtaylor.github.io/gntat14.

**Supplementary information**: Supplementary data are available at *Bioinformatics* online.

**Contact:** avigail.taylor@dpag.ox.ac.uk

## 1 INTRODUCTION

In search of the genetic causes of diseases, high-throughput experiments, such as genome-wide association and gene expression studies, are often used to find putatively causative genetic variants. Such experiments implicate many genes (‘seed-genes’); to gain insights into the biological mechanisms through which these genes’ variants exert their effects, we often explore the hypothesis that the seed-genes participate in a shared biological pathway or process. To this end, within a given biological network, (say, a protein–protein interaction (PPI) or gene co-expression network), we can count the direct connections between the seed-genes [seed-genes and direct connections between them comprise the ‘direct network’ (see Supplementary Figure S1A)], and determine if this ‘direct seed connectivity’ is more than expected by chance.

Broadly speaking, there are two ways to assess direct seed connectivity within a network of interest: In ‘seed randomization’ (SR), we keep the network the same, randomly select gene-sets equal in size to the set of seed-genes, and obtain an empirical *P*-value by comparing the direct seed connectivity to the connectivity of the random gene-sets (see Supplementary Figure S2A); conversely, in ‘network permutation’ (NP), we keep seed-genes the same, permute the edges of the network many times (while preserving node degree and network clustering structure), and obtain an empirical *P*-value by comparing the direct seed connectivity in the real versus permuted networks (see Supplementary Figure S2B and Supplementary Information for algorithmic details).

Sometimes, experimental biases influence seed-gene selection; for example, when using RNA-sequencing to call differentially expressed genes, there is a length bias towards longer transcripts (Oshlack and Wakefield, 2009). In such cases, gene attributes affected by these biases must be accounted for when assessing direct seed connectivity. In SR, we can do this by matching gene attributes of randomized gene-sets to the real set of seed-genes (see Supplementary Figure S3; note this approach will not work if seed-genes have unique attributes). In other scenarios, the biological network used to determine direct seed connectivity may be subject to an ascertainment bias; for example, gene–gene connections reported in PPIs are biased by the number of studies pertaining to processes in which gene products participate (Rossin *et al.*, 2011). With SR, we can account for an ascertainment bias by matching randomized genes to real seed-genes by degree. However, if such a bias is the primary concern, then NP should be used to better account for node degree [on condition that the network in question can be sufficiently permuted, while preserving its clustering structure (see Additional features)]. NP can also be used to evaluate three further properties of the seed-gene network [proposed in (Rossin et al., 2011), but renamed here]: ‘seed direct degrees mean’; ‘seed indirect degrees mean’; and ‘common connectors degrees mean’. [The latter two properties use the ‘indirect network’ among seed-genes (see Supplementary Figure S1B); Supplementary Figures S4–S6 explain these properties.] Last, NP can help identify which seed-genes, if any, might be hubs in the direct network (see Supplementary Figure S4).

### 1.1 Existing platforms

A popular implementation of NP is the online resource Disease Association Protein-Protein Link Evaluator (DAPPLE) (Rossin et al., 2011). Crucially, unlike other implementations of NP (Alexeyenko *et al.*, 2012; Poirel *et al.*, 2011), the DAPPLE NP algorithm preserves not only node-degree but also network clustering structure. However, DAPPLE has important limitations: its web-based interface forces users to compete with global users for resources and is a bottleneck in any high-throughput pipeline; in addition, users are constrained to use DAPPLE’s built-in PPI. GeneNet Toolbox addresses these problems: it is standalone, can be run from the command-line, and allows flexible input. Moreover, it employs a MATLAB-optimized NP algorithm that preserves both node degree and clustering structure (see Supplementary Information for a performance comparison), calculates the same network properties for lists of seed-genes as DAPPLE, and also enables additional analyses (described later).

## 2 FEATURES

Users access GeneNet Toolbox via a graphical user interface, or by calling command-line functions (see user manual available as Supplementary Information). The toolbox enables users to assess seed-gene connectivity in a user-specified network using either SR or NP. With SR users can account for gene-attributes. Two extensions are available for both methods. The first restricts an analysis to a specified sub-network of the primary biological network (a ‘background’; see Supplementary Figure S7), thus enabling the user to assess the connectivity of seed-genes within a particular genic context, rather than against a ‘whole-genome’ background. This might be useful, for example, in the analysis of a behavioural disorder, when a user might want to compare the connectivity of seed-genes to genes associated with behaviour, rather than to all genes. The second extension calculates connectivity from the initial seed-genes to a second set of interesting genes (a ‘backbone’; see Supplementary Figure S8). Thus, for example, in analysing the genetic causes of a disease, a user could assess connectivity of seed-genes to previously identified candidate-genes for that disease. The extensions can be combined (see Supplementary Figure S9). If available, multiple processors can be used to reduce run-time. To visualize the seed-gene direct network users choose the ‘quickview’ option, or output the network as a tab-delimited text file formatted for Cytoscape (Smoot *et al.*, 2011). Input files are tab-delimited text files; results files are text files.

### 2.1 Additional features

As noted above, when network ascertainment bias is of concern (e.g. in a PPI), seed-gene connectivity is likely assessed more accurately with NP than SR. However, for NP to work it must be possible to permute a network sufficiently while preserving its clustering structure. To assess a network’s suitability for NP, we provide a pre-analysis tool ‘Network permutation analyser’ (see user manual). The tool summarizes a network’s clustering structure using the global and mean local clustering coefficients, (Luce and Perry, 1949; Watts and Strogatz, 1998), comparing these attributes to those obtained for permuted networks. Heuristic measures indicating the effect of network permutations are also provided: for *N* permutations (set by the user) the tool: (i) shows the mean percentage of edges remaining unbroken per permutation; and (ii) plots the percent of edges remaining unbroken after 1..*N* permutations. Finally, we expect that networks in which hub-genes are connected to hub-genes will be hardest to permute, so to assess network assortativity we plot the neighbour connectivity distribution (Maslov and Sneppen, 2002).

## 3 PERFORMANCE

(Table 1).

**Table 1.** Computational performance of SR and NP

Network			Load time		SR (10 000 r)		NP (10 000 p*)	
Type	Genes	Edges	D	HS	D	HS	D	HS**
PPI	12 648	167 445	1.6 s	1.6 s	22 s	33 s	∼2.2 h	∼0.5 h
CO	14 449	8 482 112	59.3 s	50.5 s	29 s	38 s	∼9 d	∼45 h

For two networks [protein–protein interaction (PPI); Co-expression (CO)], we timed SR and NP with 50 seed-genes, for 10 000 randomizations (r)/permutations (p), on a desktop (D) and high-specification (HS) computer. D: 3.6-GB RAM and two 3.16-GHz Intel Core2 Duo CPUs. HS: 148-GB RAM and 24 2.67-GHz Intel Xeon CPUs.*Ten switches per permutation. **Timed using ‘parallelize’ option (see user manual).

## 4 REQUIREMENTS

GeneNet Toolbox requires MATLAB 2013B (at least) and Perl, and can be used on any computer where these are installed. Alternatively, the toolbox is available as a set of standalone applications that can be run without a MATLAB installation or license, in a Linux environment (Perl is required). The toolbox is distributed under the GNU General Public License v3.0 (http://www.gnu.org).

*Funding*: This work was supported by the Medical Research Council (AT, CW); the Wellcome Trust [093941/Z/10/Z] (JS), [090532/Z/09/Z] (The Wellcome Trust Centre for Human Genetics); the European Union’s Seventh Framework Programme project GENCODYS [241995] (CW, AT); and Somerville-Clarendon and Natural Sciences and Engineering Research Council of Canada Scholarships (TA).

*Conflict of interest*: None declared.

## Supplementary Material

Supplementary Data
