# Supplementary material for: GeneNet Toolbox for MATLAB: a flexible platform for the analysis of gene connectivity in biological networks
Source: Bioinformatics. 2014 Oct 14;31(3):442–4. doi: 10.1093/bioinformatics/btu669 (PMC4308667; doi:10.1093/bioinformatics/btu669)
Supplement: Supplementary Data [file supp_31_3_442__index.html]

GeneNet Toolbox for MATLAB: a flexible platform for the analysis of gene connectivity in biological networks — GeneNet Toolbox for MATLAB: a flexible platform for the analysis of gene connectivity in biological networks — Supplementary Data 

# GeneNet Toolbox for MATLAB: a flexible platform for the analysis of gene connectivity in biological networks

## Supplementary Data

files

**Files in this Data Supplement:**

- Supplementary Data - pdf file
- Supplementary Data - pdf file
