# Supplementary material for: GeneNet Toolbox for MATLAB: a flexible platform for the analysis of gene connectivity in biological networks
Source: Bioinformatics. 2014 Oct 14;31(3):442–4. doi: 10.1093/bioinformatics/btu669 (PMC4308667; doi:10.1093/bioinformatics/btu669)
Supplement: Supplementary Data [file supp_btu669_btu669-ATaylor_User_Manual.pdf]

GeneNet Toolbox for MATLAB: a flexible platform for the analysis of gene connectivity in biological networks: User Manual

Table of Contents

A Note on Network Nomenclature ('genes' Versus 'nodes') 4

System Requirements 5

Option 1 – Using GeneNet Toolbox in a MATLAB Environment 5

Option 2 – Using GeneNet Toolbox Tools as Standalone Applications 5

License 5

Download and Installation 6

Option 1 – MATLAB Environment 6

Option 2 – Standalone Tools 6

Quick-Start 8

Option 1 – MATLAB Environment 8

Option 2 – Standalone Tools (under Linux) 8

GeneNet Toolbox: MATLAB Command-line Functions 10

MATLAB Command-line Functions Reference Table 10

GeneNet Toolbox Standalone Tools: Linux Commands 11

Linux Commands Reference Table 11

GeneNet Toolbox – Graphical User Interface 12

MATLAB Environment 12

Standalone Tools 12

'Input' Panels 14

'Basic' Panel 14

'Background' Panel 16

‘Backbone’ Panel 16
‘Results’ Panel 16
‘Options’ Panel 17
‘Controls’ Panel 17
‘Results Summary’ Panel 18
‘Quickview’ Panel 19
Input File Formats 21
Seed File 21
Background and Backbone Files 22
Node Attribute File 22
Output Files 23
Output Files Reference Table 23
Network Permutation Analyser 26
Introduction 26
Graphical User Interface 26
MATLAB Environment 26
Standalone Tools 26
‘Input’ Panel 29
‘Network Attributes’ Panel 29
‘Nodes and Edges’ Panel 30
‘Clustering’ Panel 30
‘Assortativity’ Panel 30
‘Controls’ Panel 31
‘Permuted Network Attributes’ Panel – Clustering 31
‘Permutation Attributes’ Panel 32
Additional Notes for GeneNet Toolbox 34

Memory Problems 34

GUI and Command Line Usage Warning 34

Warning Messages 34

References 35

A Note on Network Nomenclature (‘genes’ Versus ‘nodes’)

GeneNet Toolbox provides functions for analysing the connectivity of nodes within biological networks. In these biological networks, genes are the nodes, while the biological connections between genes (for example, gene-gene co-expressions or protein-protein interactions) are the edges. In the main paper describing GeneNet Toolbox (Taylor, et al., 2014) we usually refer to genes, rather than nodes, because the focus is on the biological relevance of the tools we have provided in GeneNet Toolbox. However, here we will refer to nodes rather than genes because the focus is on the available functionality (which can, indeed, be applied to non-biological networks too). Similarly, while we usually refer to ‘seed-genes’ in the main paper, throughout this document we refer simply to ‘seeds’.

System Requirements

Option 1 – Using GeneNet Toolbox in a MATLAB Environment

Running GeneNet Toolbox in a MATLAB environment requires at least MATLAB 2013B and Perl. No compilation is required. The toolbox can be used on any computer where MATLAB and Perl are installed, and is therefore available for Windows, Linux, and MAC OS machines.

Option 2 – Using GeneNet Toolbox Tools as Standalone Applications

GeneNet Toolbox tools are also available as a set of standalone applications that can be run in a Linux environment. Perl must be installed. Running these applications requires installation of the MATLAB Compiler Runtime (version R2013b (8.2)), which is freely available from
http://www.mathworks.com/products/compiler/mcr/index.html
You do not need a MATLAB license to run these GeneNet Toolbox standalone tools. No compilation is required.

License

GeneNet Toolbox is open source and distributed under the GNU General Public License v3.0 (http://www.gnu.org).

Download and Installation

Option 1 – MATLAB Environment

GeneNet Toolbox is available from http://avigailtaylor.github.io/gntat14. Download the ZIP file or the TAR ball, unzip/ extract the download, and save the whole genenet directory (folder) in your favourite directory for your MATLAB downloads. The genenet directory contains everything you need to run GeneNet Toolbox and the Network Permutation Analyser. To install GeneNet Toolbox you need to set the MATLAB path to include the full path to where you have saved the genenet directory. The easiest way to do this is to start MATLAB, then using the MATLAB desktop:

1. In the 'HOME' tab, under 'ENVIRONMENT', click 'Set Path'.
2. In the 'Set Path' dialogue box that comes up, click the 'Add with Subfolders...' button.
3. A file selection dialogue box will appear; use this to navigate to the genenet directory. Highlight the genenet directory, and then click 'Open'.
4. Click 'Save' in the 'Set Path' dialogue box.

You are now ready to use GeneNet Toolbox!

Option 2 – Standalone Tools

GeneNet Toolbox standalone tools are available (for Linux) from http://avigailtaylor.github.io/gntat14. Download the ZIP file or the TAR ball, unzip/ extract the download, and save the genenet_standalone directory in

your favourite directory for applications. To install GeneNet Toolbox standalone tools, carry out the following steps:

1. Open a terminal
2. cd to the genenet_standalone directory
3. You need to change the permissions on all of the *.sh files, so that they can be executed: Type the following at the prompt, then press return:

   ```
   chmod +x *.sh
   ```

If you do not have a copy of the Linux 64-bit version of the MATLAB Compiler Runtime (MCR), download it from Mathworks by navigating to:
http://www.mathworks.com/products/compiler/mcr/index.html
Save the downloaded file to the genenet_standalone directory, return to the terminal opened above, and type the following at the prompt (press return after each statement):

```
unzip MCR_R2013b_glnxa64_installer.zip
cd MCR_R2013b_glnxa64_installer
./install
```

Follow the installer instructions. (**IMPORTANT**: During the installation you will be asked to choose a directory in which to install the MCR. Make a note of the full path of this directory. When you run the standalone tools you will need to know the mcr_directory. This is: full_path_to_mcr_install_directory/v82).

You are now ready to use GeneNet Toolbox standalone tools!

Quick-Start

For the following examples you will need to make a note of the full path to your copy of the genenet directory (referred to as genenet_dir below).

Option 1 – MATLAB Environment

To run a basic seed-connectivity analysis using seed randomisation:

1. Start a MATLAB session.
2. At the prompt type the following and press return:

```
[ total_nodes , dir_network , context_network , observed , expected , p_value ] =
sr_seed(‘genenet_dir/toy_example/toy_network’ , 0 , ‘genenet_dir/toy_example/toy_seeds’ , 0 , 100 , 0 , 1);
```

full path to network

no header line in network file

full path to seed file

no header line in seed file

100 randomisations

No parallelisation

‘quickview’ on

3. To see the results type each of the following and press return:

```
total_nodes

dir_network

context_network

observed

expected

p_value
```

Option 2 – Standalone Tools (under Linux)

To run a basic seed-connectivity analysis using network permutation:

1. Open a terminal
2. cd to the genenet_standalone directory
3. At the prompt type the following and press return:

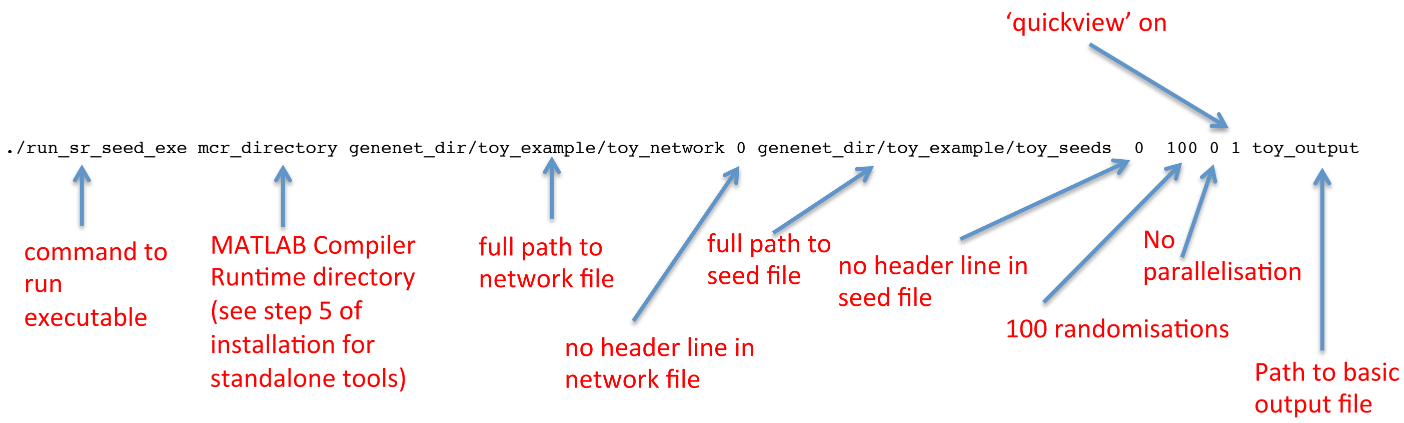

4. To view the basic results use your favourite editor to open the basic output file saved in toy_output.

GeneNet Toolbox: MATLAB Command-line Functions

MATLAB Command-line Functions Reference Table

Command	Description
**sr_seed**	Seed connectivity analysis using seed randomisation.
**sr_seed_bg**	Seed connectivity analysis using seed randomisation, and restricting the analysis to a specified sub-network (background) of the primary network.
**sr_seed2back**	Seed to backbone connectivity analysis using seed randomisation.
**sr_seed2back_bg**	Seed to backbone connectivity analysis using seed randomisation, and restricting the analysis to a specified sub-network (background) of the primary network.
**np_seed**	Seed connectivity analysis using network permutation.
**np_seed_bg**	Seed connectivity analysis using network permutation, and restricting the analysis to a specified sub-network (background) of the primary network.
**np_seed2back**	Seed to backbone connectivity analysis using network permutation.
**np_seed2back_bg**	Seed to backbone connectivity analysis using network permutation, and restricting the analysis to a specified sub-network (background) of the primary network.

To obtain detailed usage instructions type the following at the MATLAB prompt:

```
help command
```

GeneNet Toolbox Standalone Tools: Linux Commands

Linux Commands Reference Table

Command	Description
**./run_sr_seed_exe.sh**	Seed connectivity analysis using seed randomisation.
**./run_sr_seed_bg_exe.sh**	Seed connectivity analysis using seed randomisation, and restricting the analysis to a specified sub-network (background) of the primary network.
**./run_sr_seed2back_exe.sh**	Seed to backbone connectivity analysis using seed randomisation.
**./run_sr_seed2back_bg_exe.sh**	Seed to backbone connectivity analysis using seed randomisation, and restricting the analysis to a specified sub-network (background) of the primary network.
**./run_np_seed_exe.sh**	Seed connectivity analysis using network permutation.
**./run_np_seed_bg_exe.sh**	Seed connectivity analysis using network permutation, and restricting the analysis to a specified sub-network (background) of the primary network.
**./run_np_seed2back_exe.sh**	Seed to backbone connectivity analysis using network permutation.
**./run_np_seed2back_bg_exe.sh**	Seed to backbone connectivity analysis using network permutation, and restricting the analysis to a specified sub-network (background) of the primary network.

To obtain detailed usage instructions type the following at the Linux prompt:

`command --help`

GeneNet Toolbox – Graphical User Interface

MATLAB Environment

To start the GeneNet Toolbox GUI using MATLAB:

1. Open a MATLAB session.
2. At the prompt, type: `genenet_toolbox`
3. Press return.

Standalone Tools

To start the GeneNet Toolbox GUI on a Linux computer:

1. Open a terminal.
2. cd to the genenet_standalone directory.
3. At the prompt type:

   ```
   ./run_genenet_toolbox.sh mcr_directory
   ```

4. Press return.

In both cases, the following screen will appear:

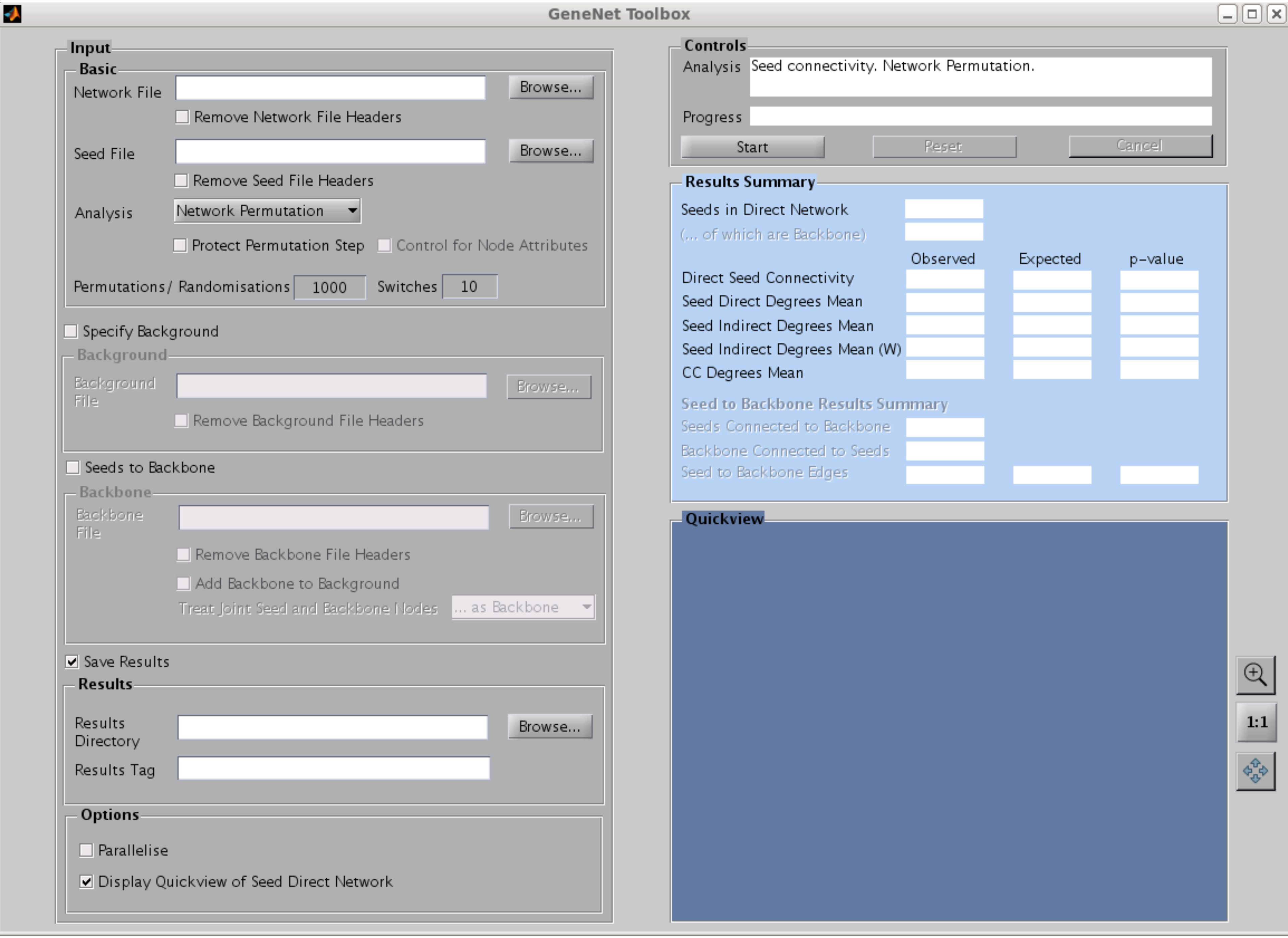
GeneNet Toolbox
Input
Basic
Network File
Browse...
Remove Network File Headers
Seed File
Browse...
Remove Seed File Headers
Analysis
Network Permutation
Protect Permutation Step
Control for Node Attributes
Permutations/ Randomisations
1000
Switches
10
Specify Background
Background
Background File
Browse...
Remove Background File Headers
Seeds to Backbone
Backbone
Backbone File
Browse...
Remove Backbone File Headers
Add Backbone to Background
Treat Joint Seed and Backbone Nodes
... as Backbone
Save Results
Results
Results Directory
Browse...
Results Tag
Options
Parallelise
Display Quickview of Seed Direct Network
Controls
Analysis
Seed connectivity. Network Permutation.
Progress
Start
Reset
Cancel
Results Summary
Seeds in Direct Network
(... of which are Backbone)
Observed
Expected
p-value
Direct Seed Connectivity
Seed Direct Degrees Mean
Seed Indirect Degrees Mean
Seed Indirect Degrees Mean (W)
CC Degrees Mean
Seed to Backbone Results Summary
Seeds Connected to Backbone
Backbone Connected to Seeds
Seed to Backbone Edges
Quickview
1:1

‘Input’ Panels

Use the ‘Input’ panels to set up your required analysis.

‘Basic’ Panel

For a basic seed connectivity analysis (analysing the direct network among seeds) using either seed randomisation or network permutation, you only need to fill in this panel.

Enter the full or relative paths to your network and seed files in the ‘Network File’ and ‘Seed File’ textboxes – you can type them in, or use the ‘Browse...’ buttons. (See the **Input File Formats** section for details of how to format these text files.) Click the relative checkboxes if your files have header lines (these need to be removed). Use the ‘Analysis’ drop-down menu to select either ‘Network Permutation’ or ‘Seed Randomisation’:

When you choose ‘Network Permutation you can choose ‘Protect Permutation Step’ if you want GeneNet Toolbox to keep track of removed edges (between the permutation step and the switching step, and also between switching steps) so that they are not re-instated as network permutation proceeds. Protecting previous permutation and switching steps *may* improve performance, but the default setting is to keep this box un-checked: it is unclear whether choosing this option will speed up permutation, in light of the fact that keeping track of removed edges can be expensive in very large networks. Previous implementations of network permutation (Rossin, et al., 2011) do not do this.

When you choose ‘Seed Randomisation’ you will have the option to tick the ‘Control for Node Attributes’ checkbox. If you do this, then when you click ‘Start’, a file selection dialogue box will appear, and you will be prompted to choose a node attribute file. (See the **Input File Formats** section for details of how to format this text file.) Having chosen a node attribute file, the following screen will appear:

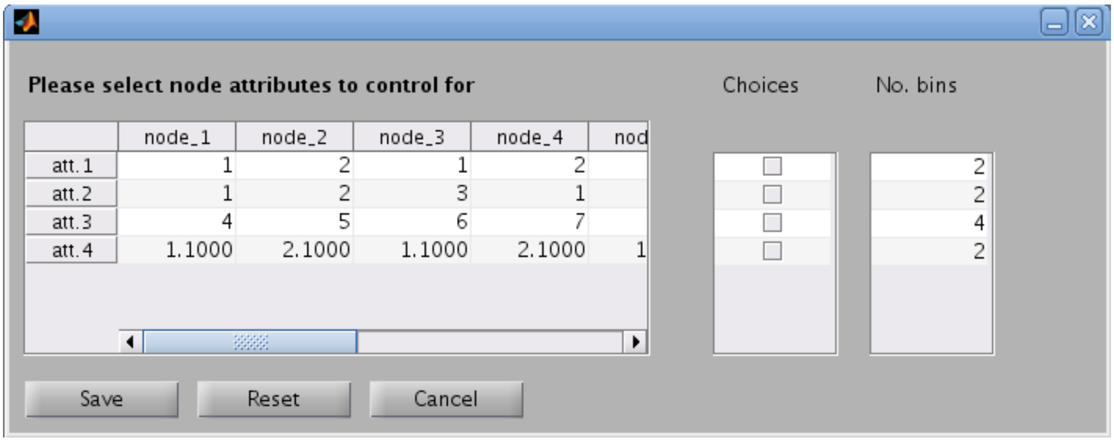

Tick the checkboxes (under ‘Choices’) for the attributes that you would like to control for and, if required, for each of the chosen attributes, edit the number of groups (‘No. bins’) to split nodes in to. (GeneNet Toolbox tries to pick the ‘best’ number of groups for each attribute[1], based on the data, but you may well want to alter these parameters).

Finally, enter the number of permutations or randomisations that you would like to perform (1000 is a good starting point), and, if using network permutation, also enter the number of switching steps to perform per permutation step.

---

[1] To implement this, we used open source code downloaded from MATLAB Central (calcnbins.m; http://www.mathworks.co.uk/matlabcentral/fileexchange/21033-calculate-number-of-bins-for-histogram/content/calcnbins.m)

‘Background’ Panel

If you want to restrict your analysis to a specified sub-network (background) of the primary network, then check the ‘Specify Background’ checkbox. This will enable the ‘Background’ panel, so that you can choose a background file (see **Input File Formats** section); once again, please tick the ‘Remove Background File Headers’ checkbox if your file has a header line.

‘Backbone’ Panel

If you want to perform a seed to backbone connectivity analysis, check the ‘Seeds to Backbone’ checkbox. This will enable the ‘Backbone’ panel, and you will be able to choose a backbone file (see **Input File Formats** section); please tick the ‘Remove Backbone File Headers’ checkbox if your file has a header line. You will need to choose how treat nodes that appear in both the seed list and the backbone list (as backbone, as seeds, or as neither); use the ‘Treat Joint Seed and Backbone Nodes’ drop-down menu to do this. If you have also chosen to restrict your analysis to a specific background, then you can add the backbone nodes to the list of background nodes by checking the ‘Add Backbone to Background’ checkbox. (For details of how these choices will affect your analysis, please see the **Supplemental Figures** accompanying (Taylor, et al., 2014)).

‘Results’ Panel

Tick the ‘Save Results’ checkbox if you would like to store the results of your analysis. Using the ‘Results Directory’ textbox, enter the relative or full path of the directory where you would like your results to be stored. Enter a results

tag into the ‘Results Tag’ textbox: this will be used as a prefix for all the results files outputted for this analysis.

‘Options’ Panel

Check the ‘Parallelise’ checkbox if your computer has multiple independent processors: you can reduce run-time by using them to run multiple permutations/ randomisations at the same time. Note that there is an overhead for setting up parallel processing and also for sharing computation among multiple processors, therefore this setting is most useful when conducting a network permutation analysis.

Check the ‘Display Quickview of Seed Direct Network’ checkbox to visualise the direct connectivity among seeds (and backbone nodes, if included), at the end of the analysis. See the **‘Quickview’ Panel** section for further details.

‘Controls’ Panel

The ‘Analysis’ textbox in the ‘Controls’ panel displays the current chosen analysis. Note that if you have entered input in a particular panel - for example a background file in the ‘Background’ panel - but you have switched off that panel later on during set-up, then that input will not be included in your analysis, and this will be reflected in the ‘Analysis’ box.

While your analysis is running, the ‘Progress’ textbox will tell you which part of the analysis has been reached, and also when it has finished. Note that permutations are counted in 10’s.

If at any point during the set-up of your analysis you want to clear all the input fields, press ‘Reset’. Press ‘Start’ to start your analysis. You do not need to press ‘Reset’ between analyses; simply enter your new inputs, and press ‘Start’. Press ‘Cancel’ to cleanly stop your analysis partway through. Please note that the ‘Cancel’ operation can take some time to respond, depending on which part of the analysis has been reached. If you cannot wait for the ‘Cancel’ button to respond, you can press CTRL+C and then press the ‘X’ in the top right corner of the GUI (thus closing the window).

‘Results Summary’ Panel

The ‘Results Summary‘ panel shows the main results for your chosen analysis. For a full explanation of these, please refer to the **Supplemental Information** and **Supplemental Figures** accompanying (Taylor, et al., 2014). In addition please note the following:

1. For all the metrics that GeneNet Toolbox calculates (direct seed connectivity, seed direct degrees mean, etc.), alongside the observed value and corresponding *P*-value (both of which are explained fully in (Taylor, et al., 2014)) we also show an expected value: this is calculated as the mean of the metric observed across all permutations/ randomisations.
2. When the user conducts a seed to backbone connectivity analysis, we additionally provide an analysis of the direct network among seeds and backbone-nodes combined. Details of how this is implemented for SR and NP are given in **Supplementary Figure S8** of (Taylor, et al., 2014).

‘Quickview’ Panel

The ‘Quickview’ panel offers a first look at the direct network among seeds (and backbone nodes, if included)[2]; note that large direct networks can take some time to draw. You can use this panel to explore the network using the zoom and pan buttons. However, to create publication-ready images we suggest using Cytoscape (Smoot, et al., 2011) – use the ‘Save Results’ option (available in the ‘Basic’ panel, see above), to save the direct network to a tab-delimited file formatted for Cytoscape (see **Output Files** section).

Here is an example of the ‘Quickview’ panel showing the direct network observed among the protein products of nine genes, in the InWeb protein-protein interaction network ((Lage, et al., 2008; Lage, et al., 2007); available from http://www.broadinstitute.org/mpg/dapple/dapple.php):

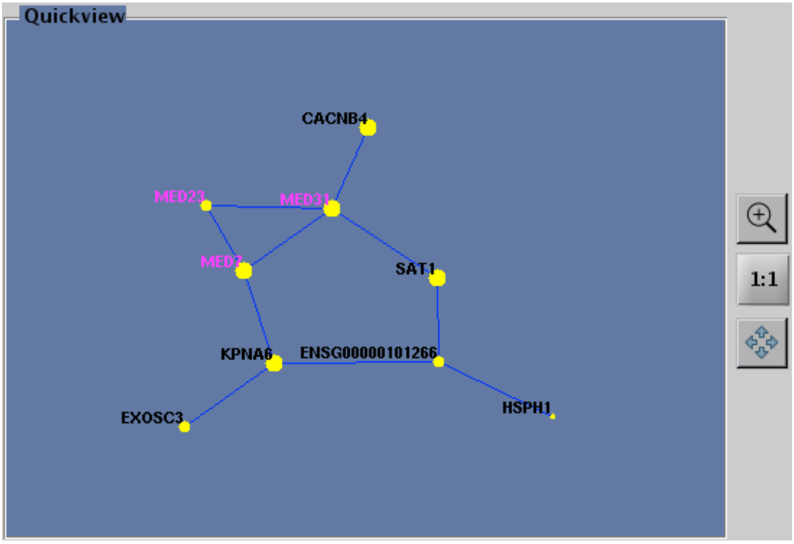

[2] To implement the ‘Quickview’ panel we used open source code downloaded from NetWiki (fruc_rein.m; http://netwiki.amath.unc.edu/VisComms/VisComms) and from MATLAB Central, (wgPlot.m; http://www.mathworks.co.uk/matlabcentral/fileexchange/24035-wgplot-weighted-graph-plot--a-better-version-of-gplot-).

The nine analysed genes comprised six seeds (labels are drawn in black), and three backbone nodes (labels are drawn in pink). Network permutation was employed for this analysis, so the size of a node (gene) reflects the probability that it is a hub in the direct network; when seed randomisation is used, all nodes are drawn the same size.

Input File Formats

Network File

- Text file listing all the edges in the network (any repeated edges will be ignored).
- Two tab- or space- delimited columns (with or without headers).
- Node names must only include alphanumeric characters (including underscore character).
- Example:

Node1	Node2
Node2	Node3
Node3	Node1
Node3	Node4
Node4	Node5
Node5	Node1

Seed File

- Text file listing all the seeds for analysis.
- One column (with or without a header).
- Node names must only include alphanumeric characters (including underscore character).
- Example:

Node3

Node1

Node2

Background and Backbone Files

- Text files listing background and backbone nodes, respectively.
- See **‘Seed File**’ for formatting and example.

Node Attribute File

- Text file tabulating node attributes to control for.
- Data should be stored in tabular format, such that columns are tab-delimited, and each row is on a new line.
- Rows are attributes and columns are nodes.
- The first row of the file must contain the node names, and the first column of the file must contain attribute names. The first element of the table (first row, first column) must be left blank.
- Node names must only include alphanumeric characters (including underscore character). Node names must be unique (that is, each node should only have one column); similarly for attribute names.
- Example:

	Node1	Node2	Node3	Node4	Node5 ...
Attribute1	x1	x2	x3	x4	x5
Attribute2	y1	y2	y3	y4	y5
Attribute3	z1	z2	z3	z4	z5

Output Files

When the user chooses to save results, a set of text files are outputted dependent on the chosen analysis. The table below lists what files are outputted for each analysis (*results_tag* is set by the user).

Output Files Reference Table

Output File	Description	Analysis *							
		1	2	3	4	5	6	7	8
results_tag.LOG	Logs the details of the analysis, including input files, parameters, node attributes controlled for, etc.	+	+	+	+	+	+	+	+
results_tag.SEEDS_PRESENT	List of seeds present in the specified/ input network, that is, the seeds that could be analysed. Note that this is not the same as the list of seeds participating in the identified direct network.	+	+			+	+		
results_tag.SEED_BB_PRESENT	List of seeds and backbone nodes present in the specified/ input network, that is, the seeds and backbone nodes that could be analysed. Note that this is not the same as the list of seeds and backbone nodes participating in the identified direct network.			+	+			+	+
results_tag.SEEDS_CONNECTED_TO_BACKBONE	List of seeds connected to backbone			+	+			+	+

	nodes.								
*results_tag.*DIRECT_CONNECTIONS	List of edges in the direct network among seeds (and backbone nodes, if provided). This file is a tab-delimited text file, with two columns, formatted for Cytoscape.	+	+	+	+	+	+	+	+
*results_tag.*INDIRECT_CONNECTIONS	List of edges in the direct network among seeds and common connectors (and backbone nodes, if provided). This file is a tab-delimited text file, with two columns, formatted for Cytoscape.					+	+	+	+
*results_tag.*CONTEXT_CONNECTIONS	List of edges in the context network. The context network is comprised of all nodes connected to a seed (or backbone node, if provided), (including seeds [and backbone nodes]), and all the edges among this set of nodes.	+	+	+	+	+	+	+	+
*results_tag.*SEED_SCORES	Empirical *P*-values for each seed's connectedness to other seeds in the direct network (*P*-values only given for seeds participating in the direct network.)					+	+		
*results_tag.*SEED_BB_SCORES	Empirical *P*-values for each seed's and each backbone-node's connectedness to other seeds and backbone-nodes in the direct network among seeds and backbone-nodes (*P*-values only given for seeds and backbone-nodes participating in the direct network among seeds and backbone-nodes).							+	+
*results_tag.*BACKGROUND_NETWORK	List of edges in the background network.		+		+		+		+

***Key for analyses:**

1. Seed connectivity analysis using seed randomisation (MATLAB command: sr_seed).
2. Seed connectivity analysis using seed randomisation, and restricting the analysis to a specified sub-network (background) of the primary network (MATLAB command: sr_seed_bg).
3. Seed to backbone connectivity analysis using seed randomisation (MATLAB command: sr_seed2back).
4. Seed to backbone connectivity analysis using seed randomisation, and restricting the analysis to a specified sub-network (background) of the primary network (MATLAB command: sr_seed2back_bg).
5. Seed connectivity analysis using network permutation (MATLAB command: np_seed).
6. Seed connectivity analysis using network permutation, and restricting the analysis to a specified sub-network (background) of the primary network (MATLAB command: np_seed_bg).
7. Seed to backbone connectivity analysis using network permutation (MATLAB command: np_seed2back).
8. Seed to backbone connectivity analysis using network permutation, and restricting the analysis to a specified sub-network (background) of the primary network (MATLAB command: np_seed2back_bg).

Network Permutation Analyser

Introduction

As explained in (Taylor, et al., 2014) some networks may be subject to an ascertainment bias, and when this is the case network permutation (rather than seed randomisation) should be used to account for node-degree while also preserving network clustering structure. The caveat to this is that network permutation does not always work, so when using network permutation on a network for the first time, you need to check that it is possible to permute the network sufficiently while preserving its clustering structure. To help assess a network's clustering structure and the efficacy of network permutations, we have provided the 'Network permutation analyser' (NPA).

Graphical User Interface

MATLAB Environment

To start the Network Permutation Analyser using MATLAB:

1. Open a MATLAB session.
2. At the prompt, type: `network_permutation_analyser`
3. Press return.

Standalone Tools

To start the Network Permutation Analyser on a Linux computer:

1. Open a terminal.
2. cd to the genenet_standalone directory.
3. At the prompt type:

```
./run_network_permutation_analyser.sh mcr_directory
```

4. Press return.

In both cases, an empty version of the following screen will appear:

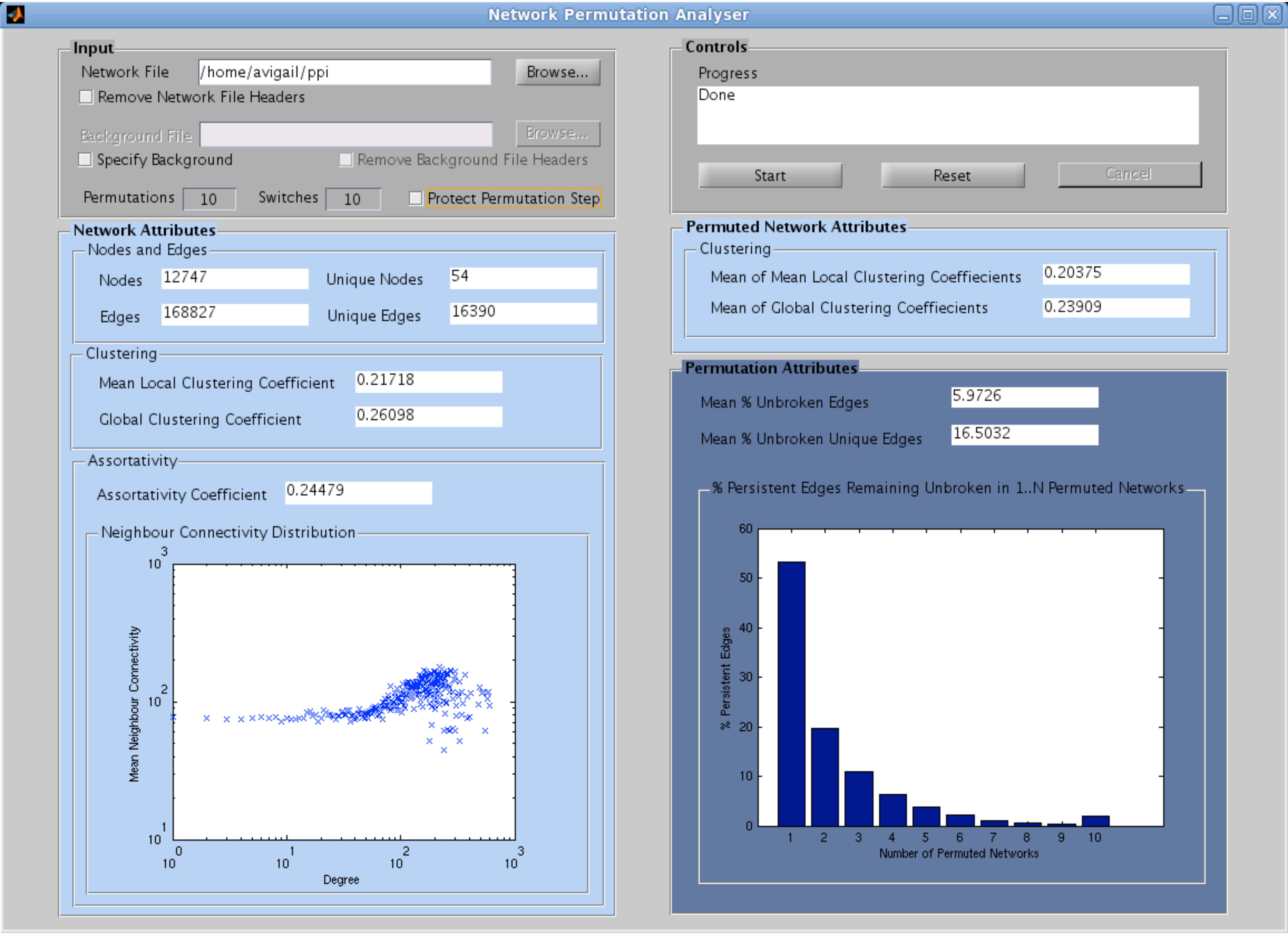
Network Permutation Analyser
Input
Network File
/home/avigail/ppi
Browse...
Remove Network File Headers
Background File
Browse...
Specify Background
Remove Background File Headers
Permutations
10
Switches
10
Protect Permutation Step
Controls
Progress
Done
Start
Reset
Cancel
Network Attributes
Nodes and Edges
Nodes
12747
Unique Nodes
54
Edges
168827
Unique Edges
16390
Clustering
Mean Local Clustering Coefficient
0.21718
Global Clustering Coefficient
0.26098
Assortativity
Assortativity Coefficient
0.24479
Neighbour Connectivity Distribution
Mean Neighbour Connectivity
Degree
Permuted Network Attributes
Clustering
Mean of Mean Local Clustering Coeffiecients
0.20375
Mean of Global Clustering Coeffiecients
0.23909
Permutation Attributes
Mean % Unbroken Edges
5.9726
Mean % Unbroken Unique Edges
16.5032
% Persistent Edges Remaining Unbroken in 1..N Permuted Networks
% Persistent Edges
Number of Permuted Networks

‘Input’ Panel

Use the ‘Input’ panel to set up your required analysis.

Enter the full or relative path to your network file in the ‘Network File’ textbox – you can type it in, or use the ‘Browse...’ button. (See the **Input File Formats** section for details of how to format this text files.)

If you want to restrict your analysis to a specified sub-network (background) of the primary network, then check the ‘Specify Background’ checkbox. This will enable the ‘Background File’ textbox, so that you can choose a background file (see **Input File Formats** section).

Click the relative checkboxes if your files have header lines (these need to be removed).

Finally, enter the number of permutations that you would like to perform (10 is good to see if network permutation is working), and the number of switching steps to perform per permutation step. Also check the ‘Protect Permutation Step’ checkbox if you are going to do this in your main analysis later on (see ‘Basic’ panel in section **GeneNet Toolbox – Graphical User Interface**, above.)

‘Network Attributes’ Panel

This part of the NPA gives you metrics pertaining to your specified network. If you have also provided a background, then the metrics are for the corresponding restricted network.

‘Nodes and Edges’ Panel

This panel shows the total number of nodes and edges in your specified network. It also shows the total number of nodes and edges in the unique sub-network of the specified network (for a definition of the unique sub-network see (Taylor, et al., 2014); **Supplemental Information**).

‘Clustering’ Panel

This panel shows the mean local clustering coefficient (Watts and Strogatz, 1998), and the global clustering coefficient (Luce and Perry, 1949), for the specified network.

‘Assortativity’ Panel

Network assortativity is of interest because it impacts the efficacy of network permutation. If you find that your network is not sufficiently permuted (see ‘Permutation Attributes’ panel, below), then the information in this panel may help explain why that is.

This panel shows the assortativity coefficient, which is the Pearson correlation coefficient of degree between pairs of linked nodes (Newman, 2002).

We also plot the neighbour connectivity distribution, defined as follows (see http://med.bioinf.mpi-inf.mpg.de/netanalyzer/): The connectivity of a node is the number of its neighbours (that is, its degree). Neighbour connectivity of a node *n* is defined as the mean connectivity, (or degree), of all neighbours of *n* (Maslov and Sneppen, 2002). Finally, the neighbour connectivity distribution

gives the average of the neighbour connectivities of all nodes *n* with *k* neighbours (that is, all nodes *n* with degree *k*) for *k* = 1, 2, ...

If your network has a large assortativity coefficient and an increasing neighbour connectivity distribution, then hub-nodes (nodes with high degree) are often connected to hub-nodes; that is, edges between highly connected nodes prevail in the network, which may make it hard (if not impossible) to permute.

'Controls' Panel

While your network permutation analysis is running, the 'Progress' textbox will tell you which part of the analysis has been reached, and also when it has finished. Note that permutations are counted in 10's.

If at any point during the set-up of your analysis you want to clear all the input fields, press 'Reset'. Press 'Start' to start your analysis. You do not need to press 'Reset' between analyses; simply enter your new inputs, and press 'Start'. Press 'Cancel' to cleanly stop your network permutation analysis partway through. Please note that the 'Cancel' operation can take some time to respond, depending on which part of the analysis has been reached.

'Permuted Network Attributes' Panel – Clustering

Here, the NPA gives you metrics pertaining to permutations of your specified network (again, if you have also provided a background, then the metrics are for permutations of the corresponding restricted network). In particular, we show the mean of the mean local clustering coefficient, and the mean of the

global clustering coefficient, obtained over all of the permuted networks. If these values are close to the clustering coefficients obtained for your specified network (see ‘Clustering’ panel in ‘Network Attributes’ panel), then this suggests that network structure is preserved during network permutation.

‘Permutation Attributes’ Panel

This part of the NPA will help you assess the efficacy of network permutations.

This panel shows the mean percentage of edges remaining unbroken over all the permutations, for both the whole of your specified network, and also just within its unique sub-network. In addition, for the set of edges remaining unbroken in at least one permutation (‘persistent edges’), we plot the percent that were unbroken in just one network, the percent that were unbroken in two networks, and so on, up to the total number of permutations tested (N = 10, in the example shown above). Thus, for the protein-protein interaction network analysed above (the InWeb network), we see that an average of ~6% of all edges remain unbroken per permutation, but that more than 50% of persistent edges are broken in 9/10 permutations of the network, and only ~2.5% remain unbroken in all 10 network permutations. In this example, therefore, network permutation is effective.

If network permutation does not work well for your chosen network, try increasing the number of switching steps per permutation, otherwise, refer to the **Assortativity** panel to see if there is evidence to suggest that your network is hard to permute. If this is the case, you will have to use seed randomisation for your analyses; further, if you are concerned that your

network may be subject to an ascertainment bias, then we suggest you match randomised seeds to real seeds according to their degree in the network, using the ‘Control for Node Attributes’ option in GeneNet Toolbox.

Additional Notes for GeneNet Toolbox

Memory Problems

We have tested GeneNet Toolbox on a Desktop Computer with 3.6 GB RAM and two 3.16 GHz Intel Core2 Duo CPU's, using a specified/ input network with ~10000 edges; under these circumstances, our tools performed in reasonable time (see (Taylor, et al., 2014)). However, please be aware that working with networks larger than this will require larger RAM, otherwise GeneNet Toolbox will abort with memory allocation problems. In general, when working with larger networks using a computer with limited RAM, you may also find that turning other applications off will improve performance.

GUI and Command Line Usage Warning

Using the MATLAB command line while a GUI is running will break the GUI.

Warning Messages

When running GeneNet Toolbox, either via the GUI or command-line, please be aware that warning messages are printed to the MATLAB terminal. These messages give you important, but not critical, information about your analyses. So, for example, seeds and backbone-nodes not present in your input network, and therefore omitted from your analysis, will be listed here.

References

Lage, K., *et al.* (2008) A large-scale analysis of tissue-specific pathology and gene expression of human disease genes and complexes, *Proc. Natl. Acad. Sci. U. S. A.*, **105**, 20870-20875.

Lage, K., *et al.* (2007) A human phenome-interactome network of protein complexes implicated in genetic disorders, *Nat. Biotechnol.*, **25**, 309-316.

Luce, R.D. and Perry, A.D. (1949) A method of matrix analysis of group structure, *Psychometrika*, **14**, 95-116.

Maslov, S. and Sneppen, K. (2002) Specificity and stability in topology of protein networks, *Science*, **296**, 910-913.

Newman, M.E. (2002) Assortative mixing in networks, *Phys. Rev. Lett.*, **89**, 208701.

Rossin, E.J., *et al.* (2011) Proteins encoded in genomic regions associated with immune-mediated disease physically interact and suggest underlying biology, *PLoS Genet*, **7**, e1001273.

Smoot, M.E., *et al.* (2011) Cytoscape 2.8: new features for data integration and network visualization, *Bioinformatics*, **27**, 431-432.

Taylor, A., *et al.* (2014) GeneNet Toolbox for MATLAB: a flexible platform for the analysis of gene connectivity in biological networks, *Bioinformatics,* doi: 10.1093/bioinformatics/btu669

Watts, D.J. and Strogatz, S.H. (1998) Collective dynamics of 'small-world' networks, *Nature*, **393**, 440-442.
