# Supplementary material for: GeneNet Toolbox for MATLAB: a flexible platform for the analysis of gene connectivity in biological networks
Source: Bioinformatics. 2014 Oct 14;31(3):442–4. doi: 10.1093/bioinformatics/btu669 (PMC4308667; doi:10.1093/bioinformatics/btu669)
Supplement: Supplementary Data [file supp_btu669_btu669-ATaylor_Supplementary_Info_and_Figures.pdf]

GeneNet Toolbox for MATLAB: a flexible platform for the analysis of gene connectivity in biological networks: Supplementary Information

A note on network nomenclature (‘genes’ versus ‘nodes’)

GeneNet Toolbox provides functions for analysing the connectivity of nodes within biological networks. In these biological networks, genes are the nodes, while the biological connections between genes (for example, gene-gene co-expressions or protein-protein interactions) are the edges. In the **main text** we usually refer to genes, rather than nodes, because the focus is on the biological relevance of the tools we have provided in GeneNet Toolbox. However, in the **Supplementary Information** and **Figures** we will refer to nodes rather than genes because the focus is on the implementation of network analysis functions (which can, indeed, be applied to non-biological networks too). Similarly, while we usually refer to ‘seed-genes’ in the main text, we refer simply to ‘seeds’ throughout the **Supplementary Information** and **Figures**.

Network Permutation (NP)

In the following sections we provide the pseudo code for two algorithms for permuting (edges in) networks: the first is a basic implementation described in (Rossin, et al., 2011); the second is optimised for MATLAB, and is the method we provide in GeneNet Toolbox. (For a diagrammatic explanation of NP see **Supplementary Figure S2B**).

Pseudo-code for NP – Basic implementation

The following pseudo-code is paraphrased from the Supplementary Methods available with (Rossin, et al., 2011):

`Define a network G, with n nodes and E edges.`

`Let` G_0 `be a random network with the same set of nodes as G and randomly assigned edges such that the degree of each node is the same in` G_0 `as it is in G.`

The algorithm for generating G_0 from `G` involves a permutation step and a switching step.

PERMUTATION STEP: Let k_1, k_2, k_3,..., k_m be the sequence of all possible node degrees in G and let K_i be the set of nodes that have degree k_i with $1 \leq i \leq m$. Then:

1. Repeat the following 1000 times:
   a. For every set K_i, $1 \leq i \leq m$:
      i. Choose randomly two nodes a and b from set K_i.
      ii. Swap their positions in G.

Note that in step a(ii), swapping the positions of randomly chosen nodes a and b is the same as re-wiring G such that all edges previously connected to a are now connected to b, and vice-versa. Thus, the effect of step 1 is to permute edges in G. However, note also that this permutation step cannot perturb nodes that have unique degrees. For this, perform the switching step on G after the permutation step.

SWITCHING STEP: Define G_{unique} to be the set of nodes with unique degrees and their edges. Let E_{unique} be the set of edges in G_{unique} and apply the following to G_{unique}:

1. Repeat the following $T_2|E_{unique}|$ times (where T_2 is the switching threshold, chosen large enough that the mixing is sufficient):
   a. While there are nodes unvisited:
      i. Choose randomly edges A-B and C-D.
      ii. If edges A-D and B-C do not exist:
         1. Add edges A-D and B-C to G_0.
         2. Remove edges A-B and C-D from G_0.
      iii. Mark nodes A, B, C and D as visited.

This algorithm creates permuted networks perfectly matched for size, binding degree of proteins and overall network structure.

Pseudo-code for NP – MATLAB-optimised implementation

In MATLAB, a direct implementation of the above pseudo-code would be too inefficient to run in reasonable time. Therefore, in GeneNet Toolbox, we optimised the algorithm for MATLAB by identifying where data could be stored as matrices, and converting as much of the implementation as possible to use built-in matrix-based functions.

Define a network G, with n nodes and E edges.

Represent G as a non-weighted adjacency matrix G_m.

Let G_0 be a random network with the same set of nodes as G and randomly assigned edges such that the degree of each node is the same in G_0 as it is in G.

Represent G_0 as a non-weighted adjacency matrix G_{0m}. Initialise G_{0m} as G_m.

OPTIMISED PERMUTATION STEP: Let k_1, k_2, k_3,..., k_m be the sequence of all possible node degrees in G and let K_i be the set of nodes that have degree k_i with $1 \le i \le m$. Then:

1. For every set K_i, $1 \le i \le m$:
   a. If $|K_i| > 1$, then:
      i. Let I_{Ki} be the indices, in matrix G_m, of the nodes in K_i.
      ii. Permute rows and columns corresponding to I_{Ki} in matrix G_{0m}. (Use the same re-

ordering permutation for rows and columns).

As for the ordinary permutation step described above, this optimised permutation step cannot perturb nodes that have unique degrees. For this, perform the optimised switching step on G_{0m} after the optimised permutation step.

OPTIMISED SWITCHING STEP: Define G_{unique} to be the set of nodes with unique degrees and their edges. Let E_{unique} be the set of edges in G_{unique}. Then:

1. Repeat the following S (default 10) times[a], until mixing is sufficient:
    a. Let RandomPairs be a matrix with $\lfloor |E_{unique}|/2 \rfloor$ rows and 4 columns.
    b. Populate RandomPairs such that each row contains a randomly chosen edge-pair from E_{unique} (columns 1 and 2 containing nodes 1 and

[a] Each optimised switching step switches many edge-pairs at once (compared to just one edge-pair switch per ordinary switching step); this is why only a relatively small number of optimised switching steps are required, compared to the number of ordinary switching steps required for the same level of mixing. The effect of changing S can be heuristically assessed using the Network permutation analyser tool (see the **User Manual** for details).

2 of the first edge, and columns 3 and 4 containing nodes 1 and 2 of the second edge).

At this point, each edge occurs in at most one pair (if there are an odd number of edges in E_{unique} then one edge will not be in RandomPairs).

c. Check for pairs of edges that share at least one node. These edges cannot be switched. Try and replace these pairs of edges with pairs that can be switched: For 100 attempts, or until all pairs of edges are switchable:
   i. For each row of RandomPairs containing an un-switchable edge-pair, keep the first edge the same (i.e. columns 1 and 2), and replace the second edge (i.e. columns 3 and 4), with a randomly chosen edge from E_{unique}.

After this step, some edges may appear more than once in RandomPairs.

d. Create a matrix, NewEdgePairs, as follows:
   i. Switch half the edge-pairs (chosen at random) in RandomPairs by re-ordering the columns to 1 3 2 4.

Thus, an edge-pair consisting of edges A-B and C-D will become an edge-pair consisting of A-C and B-D.

ii. Switch the other half of edge-pairs by re-ordering the columns to 1 4 2 3.

Thus, an edge-pair consisting of edges A-B and C-D will become an edge-pair consisting of A-D and B-C.

e. Remove any edge-pairs from NewEdgePairs where one or both of the edges already exist in E_{unique}.

Every edge-pair that is formed requires two existing edges to be broken, but existing edges can only be broken once. Therefore:

f. Filter NewEdgePairs as follows:
   i. Create a matrix, BrokenEdgePairs by re-ordering the columns of NewEdgePairs as 1 3 2 4.

So, if edge-pair A-B and C-D were previously switched to A-C and B-D, they will be re-ordered to A-B and C-D; and if they were switched to A-D and B-C, they will be re-ordered to A-B and D-C (which is the same as edge C-D).

ii. Only keep rows in NewEdgePairs for which the two edges in the same row of BrokenEdgePairs appear for the last[b] time in BrokenEdgePairs.

Each new edge-pair can only be created once, therefore:

g. Only keep rows in NewEdgePairs for which both edges are the last[c] instance of themselves in the whole of NewEdgePairs.
h. Add edges in NewEdgePairs to G_{0m}.
i. Create matrix EdgesToRemove by re-ordering the columns of NewEdgePairs to 1,3,2,4. Remove edges in EdgesToRemove from G_{0m}.

Performance Comparison for NP

We compared the performance of our NP algorithm to two similar network permutation algorithms (Alexeyenko, et al., 2012; Poirel, et al., 2011), both of which preserve node-degree, but not network clustering structure:

In their publication, Alexeyenko et al., state that one permutation of a network with ~16000 nodes and ~1.5 M edges takes ~60 seconds on a 3 GhZ PC with

[b] Ideally the rows to keep would be chosen at random, but this would make implementation too slow. As such, our choice always to keep rows in `NewEdgePairs` for which the corresponding broken edges appear for the *last* time in `BrokenEdgePairs` is arbitrary.
[c] Same argument as given in footnote b.

4 Gb RAM. We compared our algorithm to theirs using the performance measures shown for the co-expression network in Table 1 of our paper: We estimate that one permutation of a network with ~14500 nodes and ~8.5 M edges would take ~160 seconds on a similar computer[d] (so ~2.5 times as long for ~5.5 times the number of edges). In addition, unable to install the package presented by Poirel et al., we contacted the authors and asked for their performance statistics. They suggested that one permutation of a network with ~10000 genes and ~150000 edges would take <1 second on a desktop computer; similar to the time taken to permute the PPI network (~12500 nodes and ~165000 edges) tested in our manuscript.

[d] Permuting the co-expression network 10000 times takes 9 days (~780000 seconds), so one permutation takes ~78 seconds. However, our desktop had two CPUs, so we estimate that one permutation would take twice as long (~160 seconds) on the desktop used by Alexeyenko et al.

GeneNet Toolbox for MATLAB: a flexible platform for the analysis of gene connectivity in biological networks: Supplementary Figures

A note on the calculation of empirical P-values

In **Supplementary Figures S2**, **S4**, **S5**, **S6** and **S8**, we explain how we calculate the network properties introduced in the main text, and then we use semi-formal language to explain how we derive empirical *P*-values for them. In particular, we often make a statement such as: "An empirical *P*-value for property *M* is calculated by comparing *M* obtained for seeds in the real network to the value of *M* calculated for seeds in the permuted networks". The more formal construction of this is just: Let M_i be the value of *M* calculated for each permutation *i* of the network, where 1≤*i*≤N, and N is the number of permutations. Then the empirical *P*-value for *M* in the real network is:

$$\frac{\left(\sum_{i=1}^{N} S \left| \begin{matrix} 1: M_i \geq M \\ 0: otherwise \end{matrix} \right. \right) + 1}{N+1}$$

Note that we add 1 to the numerator and denominator because we also count the calculation of *M* in the real network. Also note that in **Supplementary Figures S2A** and **S8**, (when we refer to the seed randomisation algorithm), we compare *M* calculated for seeds in the network to *M* obtained for randomised seeds in the network, (that is, keeping the network the same), rather than comparing *M* obtained for seeds in the real network to *M* obtained for the same set of seeds in permuted networks; however, if we choose

randomised seed sets *N* times, then the construction of the empirical *P*-value remains as above.

Supplementary Figure S1: The direct and indirect networks among seeds.

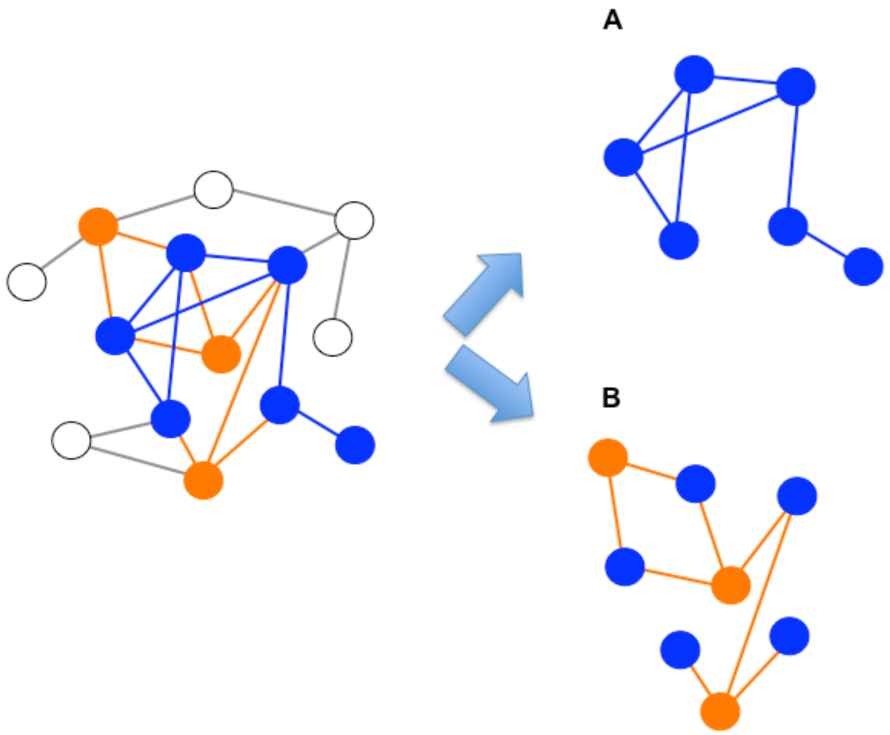

How we derive, from a whole network, the direct network among seeds, common connectors and the indirect network among seeds. On the left side of the figure we depict an example network; six seeds, and the edges connecting them, are highlighted in blue. Drawn in orange are non-seed nodes that are connected to more than one seed, termed 'common connectors'; the edges between common connectors and seeds are drawn in orange. On the right side of the figure, pointed to by blue arrows, we show the resultant direct network amongst seeds (**A**), and the resultant indirect network amongst seeds and common connectors (**B**). The direct network consists only of seeds and the edges connecting them. The indirect network consists of

common connectors, seeds connected to common connectors, and all edges connecting a seed to a common connector.

Supplementary Figure S2: The two main methods implemented in GeneNet Toolbox that test direct seed connectivity; simple examples.

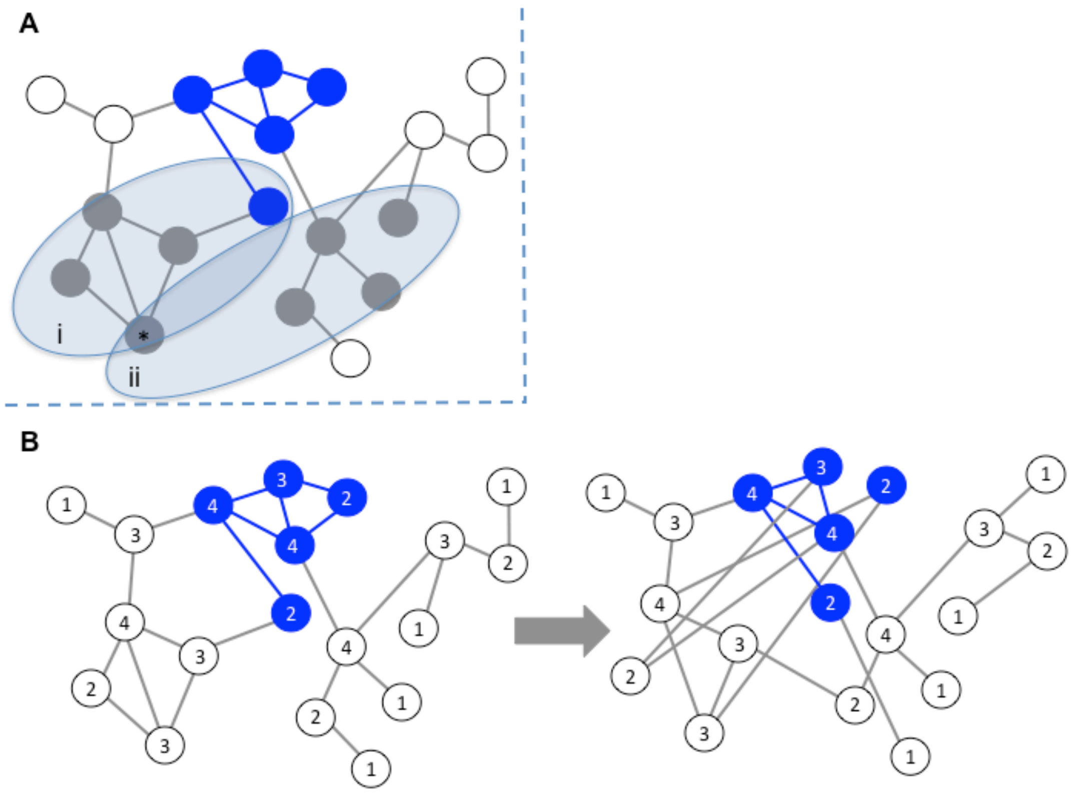

(A) ‘Seed randomisation’ (SR). We depict a small example network in which 5 of the nodes are seeds; these are drawn in blue, as are the edges connecting them. Two randomisations are highlighted as grey ellipses (i and ii). In each randomisation, five nodes in the network are randomly chosen, (depicted in grey). An empirical *P*-value for the observed direct seed connectivity is calculated by comparing the number of edges connecting the seeds to the number of edges connecting the randomly chosen nodes in each randomisation. Randomisations can include seeds (as in i), and may also include nodes chosen in previous randomisations, as is the case for the node highlighted with ‘*’ (chosen in i and ii). **(B) ‘Network permutation’ (NP).** We depict the same network shown in **(A)**; for clarity, we have also labeled nodes with their degree. One permutation of the edges in the network is shown;

edges have been permuted such that the degree of all the nodes is maintained. An empirical *P*-value for direct seed connectivity is calculated by comparing the total number of edges connecting the seeds in the real network to the total number of edges connecting them in each permuted network.

Supplementary Figure S3: Matching randomised node-set attributes to seed attributes.

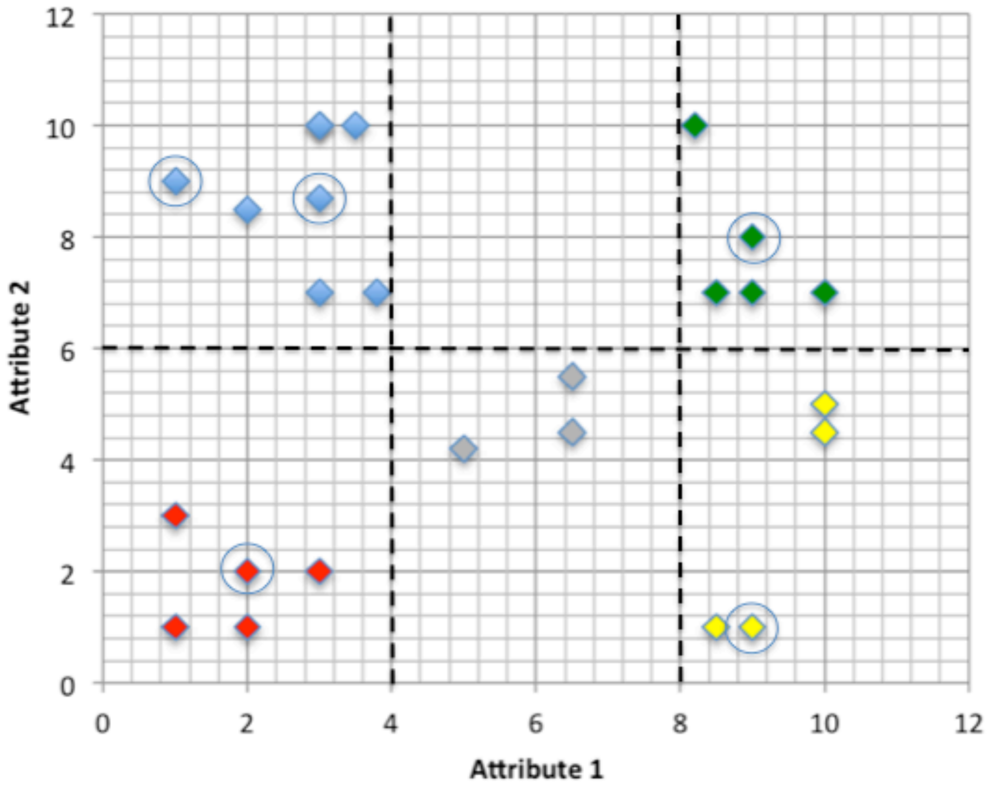

How we choose randomised sets of nodes such that the distribution of their attributes matches that of the real set of seeds. Here we depict a plot of the attribute values for 24 nodes participating in an example network, and for which two attributes have been measured for each node. There are 5 seeds, which have been circled in the plot. To choose randomised node-sets matched for attributes to these seeds, we first split the attribute space into bins, and then group (or label) nodes according to the bin that they lie in. Finally, to generate a randomised node-set, we choose the same number of random nodes from each group as there are seeds in that group. In this example, by dividing Attribute 1 in to three bins, and Attribute 2 in to two bins,

we have divided the total attribute-space into six bins. The nodes fall in to five of these bins, and are grouped accordingly (here we have labelled groups of nodes with a colour: blue; green; red; grey; or yellow). We see that the blue group contains two seeds, and that the green, red and yellow groups contain one seed each. Therefore, when picking randomised node-sets we will pick two nodes at random from the blue group, and one node at random from each of the green, red and yellow groups; no random nodes will be chosen from the grey group because there are no seeds in that group. Note that although we have used two attributes to explain our algorithm for matching (by attribute) randomised node-sets to a set of seeds, the algorithm is, in principle, generalisable to any number of attributes (the limiting factor being over-fitting of the attribute space to the nodes). Note also that the number of bins that each attribute is split in to can be chosen by the user, or instead automatically set using algorithms that identify an ‘ideal’ number of bins for a given set of data (Freedman and Diaconis, 1981; Scott, 1979; Sturges, 1926).

Supplementary Figure S4: Calculating the seed direct degrees mean and identifying potential hubs in the direct network.

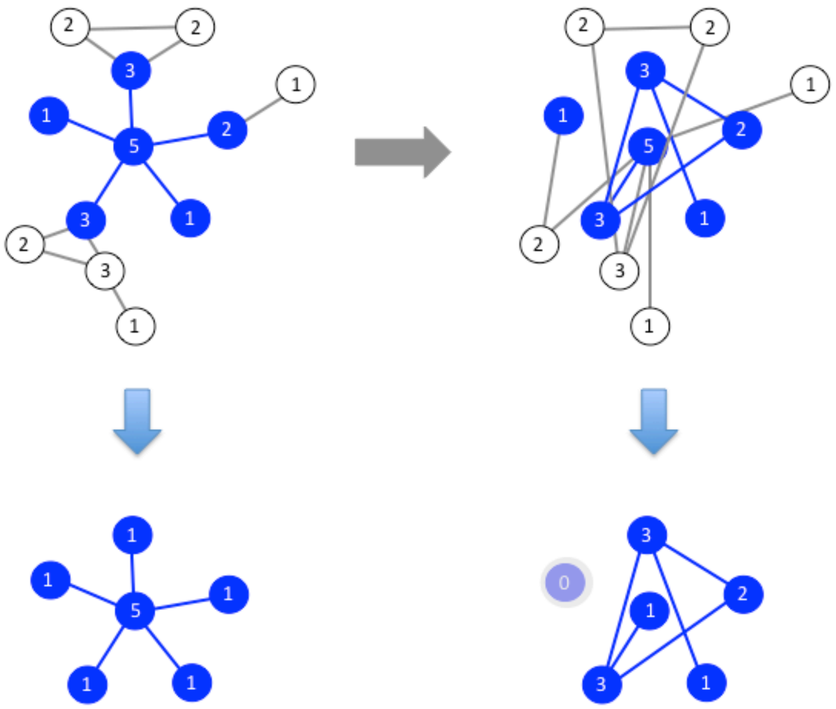

In the top left corner of the figure we depict a small example network; six seeds and the five edges connecting them are highlighted in blue. For clarity, all nodes are annotated with their degree. In the top right of the figure, pointed to by a grey arrow, we depict one possible permutation of the original network; we see that the edges in the network have been re-wired, but that the degree of each node, within the whole network, remains the same. In the bottom half of the figure, pointed to by blue arrows, we show the direct networks consisting of only seeds and their connecting edges within the original network (left), and the permuted network (right). In these direct networks seeds are annotated with their degree in the direct network, rather than the overall network. (Note that seeds with a degree of zero are, by definition, not

in the direct network; however for clarity we have drawn them here shaded in grey). In contrast to the overall network, in which seed degree remains the same after permutation, the seed degree changes in the permuted direct network. After calculating the average of the seed degrees in the original direct network (termed the ‘seed direct degrees mean’[e]), we obtain an empircal *P*-value for this property by comparison to the average seed degree observed in permuted direct networks. In addition, we can assess whether each seed has more links to other seeds than expected by chance, and hence identify potential hubs in the network. To do this we calculate a *P*-value for each seeds’ connectedness to other seeds, by comparing its degree in the real direct network to its degree in permuted networks.

---

[e] Termed ‘associated protein direct connectivity’ in (Rossin, et al., 2011).

Supplementary Figure S5: Calculating the seed indirect degrees mean using NP and the indirect seed network.

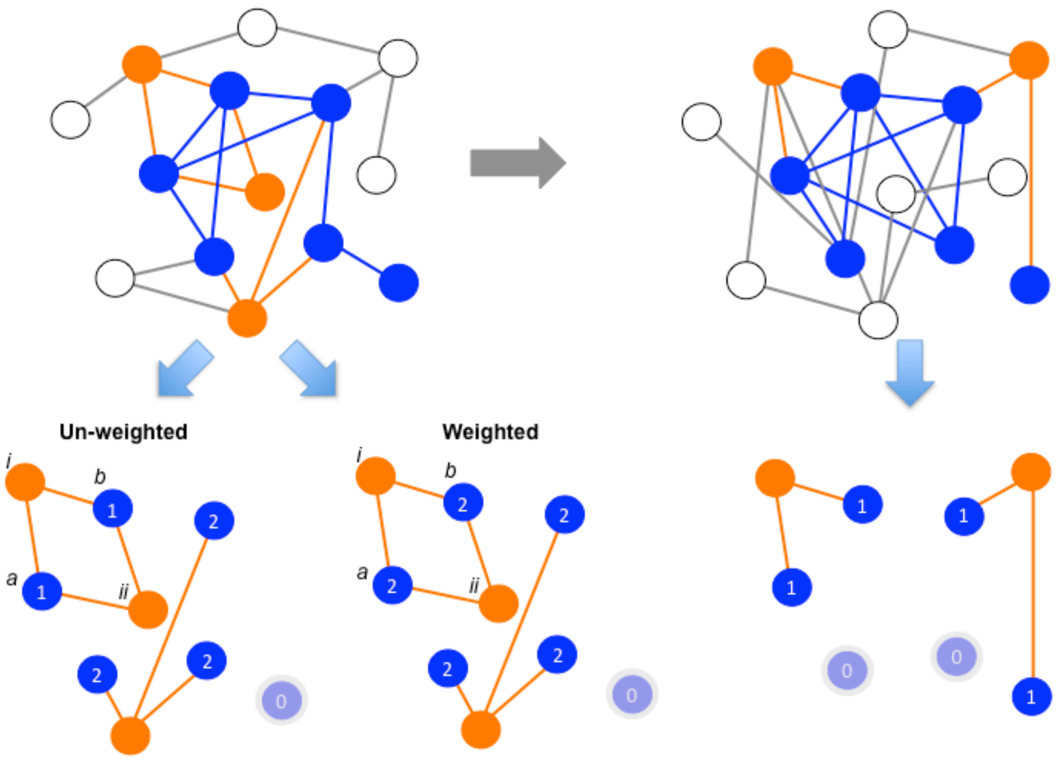

In the top left corner of the figure we depict an example starting network; as before, seeds are drawn in blue, common connectors are drawn in orange, edges connecting two seeds are in blue, and edges connecting seeds and *common connectors* are shown in orange. To the right of this network, pointed to by a grey arrow, we depict one possible permutation of the edges in the starting network. Beneath both the original and permuted networks we show the corresponding indirect networks (see **Supplementary Figure S1B**). In these indirect networks we have annotated the seeds with their degree in the indirect network (note that non-participating seeds, with a degree of zero, are shaded in grey). For a given seed, its degree in the indirect network can refer to the number of other seeds to which it is connected via one or more

common connectors (unweighted). Alternatively, the degree of a given seed can also include information about the number of common connectors that connect it to other seeds (weighted). As an example, refer to the indirect seed networks for the starting network. Here, two seeds, '*a*' and '*b*', are connected to one another via two common connectors, '*i*' and '*ii*'. In the unweighted indirect network we only count the indirect connection between *a* and *b* once, so both nodes have an indirect degree of 1; conversely, in the weighted indirect network we count both indirect connections between *a* and *b*, via *i* and *ii*, so the weighted indirect degree for both seeds is 2. Having calculated the degree for each seed in the indirect network (either unweighted or weighted), we can calculate the average of the seed degrees in the original indirect network (termed the 'seed indirect degrees mean'[f]) and obtain an empircal *P*-value for this property by comparison to the average seed degree observed in permuted indirect networks.

---

[f] Termed 'associated protein indirect connectivity' in (Rossin, et al., 2011).

Supplementary Figure S6: Calculating the common connectors degrees mean using NP and the indirect seed network.

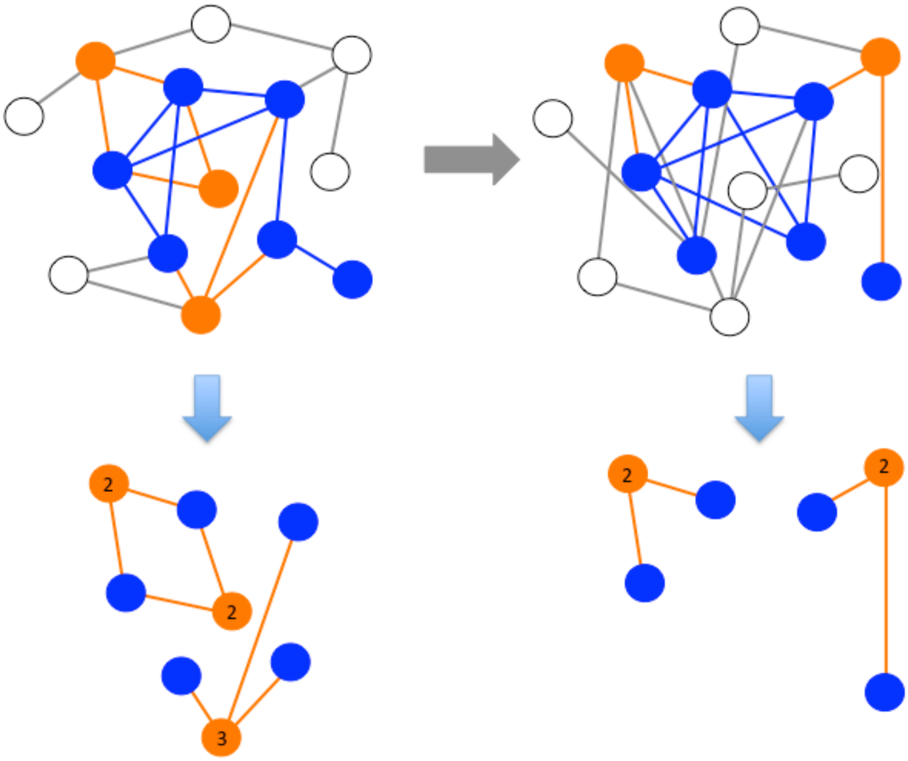

In the top left corner of the figure we depict an example starting network; as before, seeds are drawn in blue, common connectors are drawn in orange, edges connecting two seeds are in blue, and edges connecting seeds and *common connectors* are shown in orange. To the right of this network, pointed to by a grey arrow, we depict one possible permutation of the edges in the starting network. Beneath both the original and permuted networks we show the corresponding indirect networks (see **Supplementary Figure S1B**). In these indirect networks we have annotated the common connectors with their degree in the indirect network, which is just the number of seeds that they connect. We calculate the average degree of the common connectors in the

original indirect network (termed the ‘common connector degrees mean’[g]), and obtain an empircal *P*-value for this property by comparison to the average common connector degrees observed in permuted direct networks.

---

[g] Termed ‘common interactor connectivity’ in (Rossin, et al., 2011).

Supplementary Figure S7: Extension 1 - Restriction to a background.

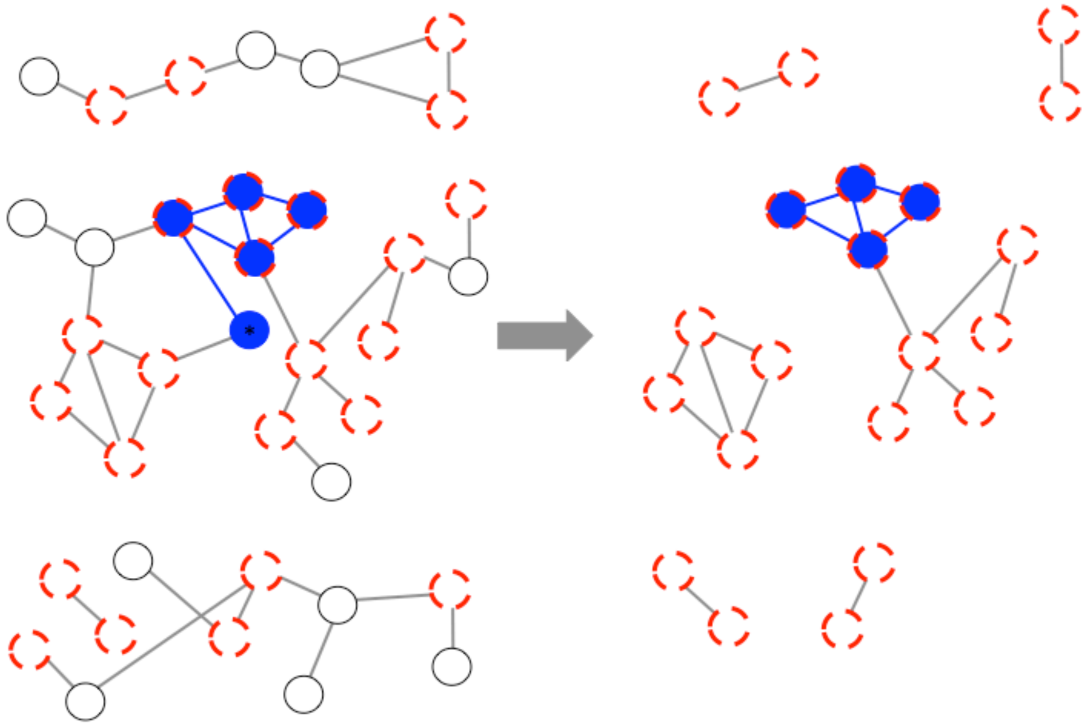

How we restrict an analysis, based on either SR or NP, to a background network specified as a node-list. On the left side of the figure we show an example starting network, with seeds and the edges connecting them drawn in blue, and nodes in the specified background node-list outlined with a dashed red line. On the right hand side we show the resultant network when nodes are restricted to the specified background, and edges are restricted to those connecting two background nodes. Note that one of the seeds is not in the specified background (highlighted with '*'), so it is not present in the restricted network.

Supplementary Figure S8: Extension 2 - Seed to backbone connectivity.

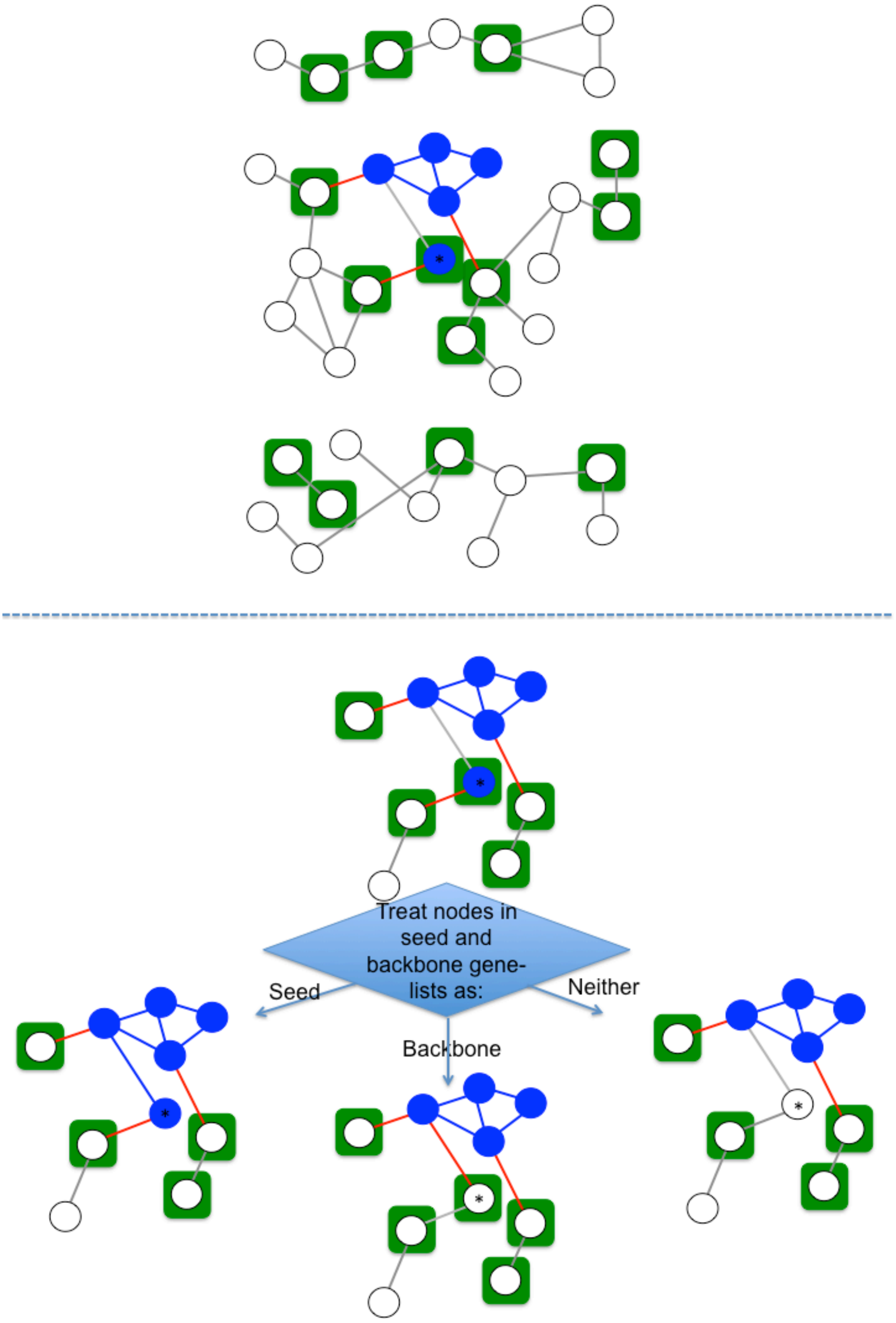

How we calculate connectivity from seeds to a specified backbone node-list.* **Top panel:** We depict an example network, with seeds and the edges connecting them drawn in blue, and nodes in the backbone node-list highlighted in green squares. One node, highlighted with a '*', is both a seed and backbone-node (see **bottom panel** for how this is dealt with). Edges that unambiguously connect a seed with a backbone-node are drawn in red. For this type of analysis, edges that connect the seeds to backbone-nodes are counted, then an empirical *P*-value is obtained by: for SR, comparing this observed total to the number of edges connecting randomly selected nodes to backbone-nodes[h] after each randomisation; for NP, comparing the observed total to the number of edges connecting seeds to backbone-nodes, after each network permutation. **Bottom panel:** As shown in the **top panel**, sometimes a node can be both a seed and backbone-node; GeneNet Toolbox enables the user to decide how to treat these nodes: as seeds; as backbone; or as neither. Here, a flowchart of the resultant networks, dependent on this decision, is shown (we have zoomed in on just that part of the network containing the affected node).

* Note that in GeneNet Toolbox, when the user conducts a seed to backbone connectivity analysis, we additionally provide an analysis of the direct network among seeds and backbone-nodes. For both SR and NP we begin by concatenating the lists of seeds and backbone-nodes (removing duplicates), and constructing a direct network as described in **Supplemental Figure S1**. Then, for SR, an empirical *P*-value for the observed direct seed and

[h] Backbone-nodes are excluded from the randomisations, so that sets of randomised nodes never include backbone-nodes.

backbone-node connectivity is calculated by comparing the number of edges among seeds and backbone-nodes to the number of edges among randomly chosen nodes and backbone-nodes in each randomisation; importantly, only seeds are randomised, while backbone-nodes are kept the same in all randomisations. For NP, an empirical *P*-value for direct seed and backbone-node connectivity is obtained by treating backbone-nodes as seeds and proceeding as described in **Supplemental Figure S2B**. Finally, when using NP for a seed to backbone analysis, the commensurate additional statistics (seed direct degrees mean, seed indirect degrees mean, and common connectors degrees mean) are obtained for seeds and backbone-nodes by treating backbone-nodes as seeds, and proceeding as described in **Supplemental Figures S4** to **S6**.

Supplementary Figure S9: Combining extensions 1 and 2.

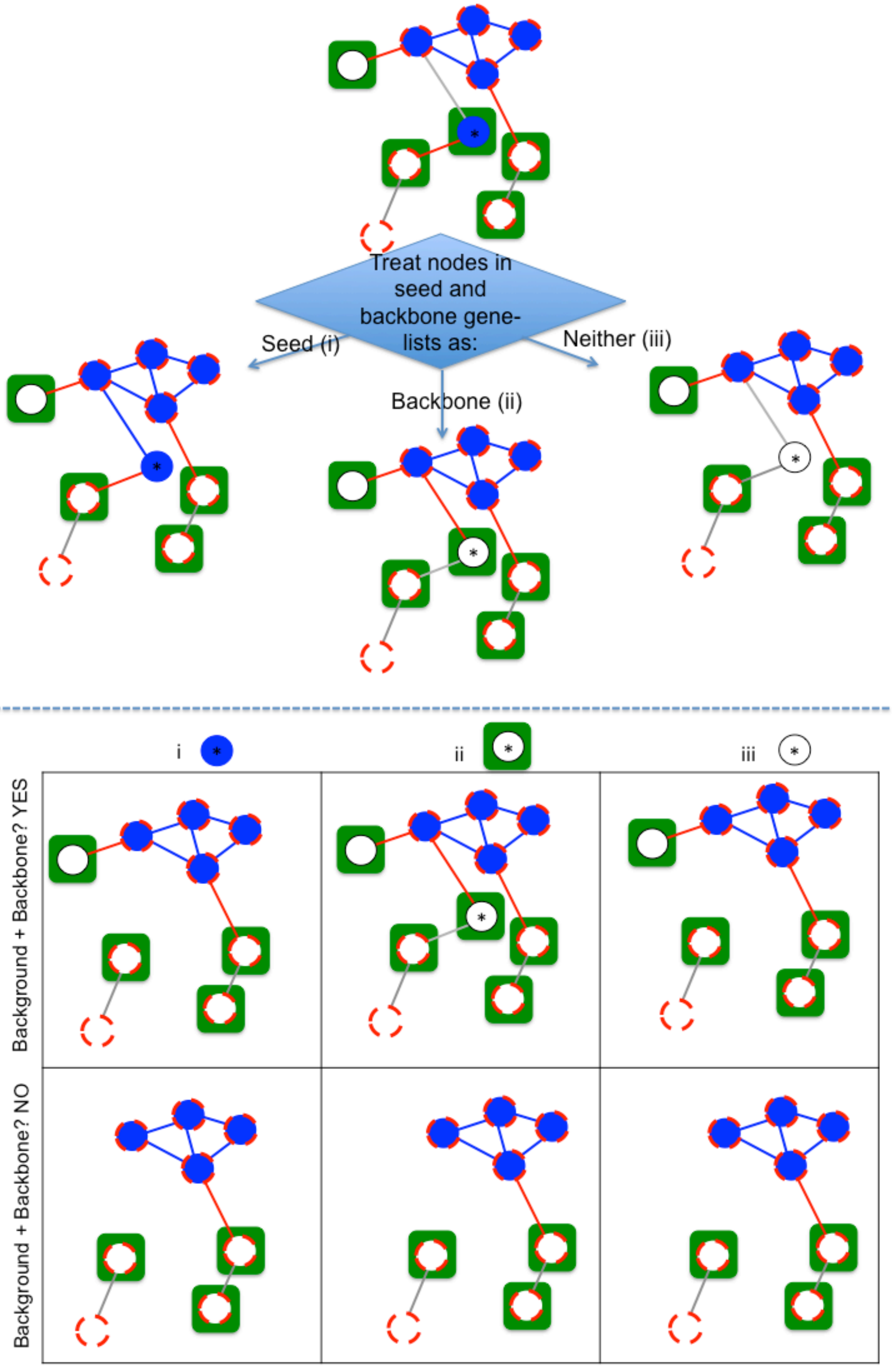

How the background and backbone extensions are combined in GeneNet Toolbox. In GeneNet Toolbox, when the user combines background and backbone analyses, the order in which analytical steps are carried out is always the same: (i) Nodes that are both seeds and backbone-nodes are dealt with (according to user input); (ii) The backbone node-list is or is not added to the background node-list (again, according to user input); (iii) The network is restricted to the updated (due to steps i and ii) background node-list; (iv) The seed to backbone analysis is conducted within the restricted network.

Here we provide a schematic depiction of the work-flow, for a simple example network, when background and backbone extensions are combined.

Top panel: We refer to the network depicted in **Supplementary Figure S8, top panel**, zooming in on the part of the network containing a node present in both the seed and backbone node-lists (highlighted with '*'), and additionally highlighting nodes present in a background node-list. Here we show a flow-chart of the resultant networks when the user chooses to treat seeds/ backbone-nodes as: (i) seeds; (ii) backbone; (iii) neither. (As before, seeds and the edges connecting them are drawn in blue, nodes in the specified background node-list are outlined with a dashed red line, nodes in the specified backbone node-list are highlighted in green squares, and edges that unambiguously connect a seed with a backbone-node are drawn in red.).
Bottom panel: Using the resultant networks from the **top panel** as input, we show a graphic table (resultant networks from **top panel** are columns) of the

final restriced network, dependent on whether the backbone node-list is added to the background node-list (rows).

References

Alexeyenko, A., *et al.* (2012) Network enrichment analysis: extension of gene-set enrichment analysis to gene networks, *BMC Bioinformatics*, **13**, 226.

Freedman, D. and Diaconis, P. (1981) On the histogram as a density estimator: L2 theory, *Zeitschrift fr Wahrscheinlichkeitstheorie und verwandte Gebiete*, **57**, 453-476.

Poirel, C.L., Owens, C.C., 3rd and Murali, T.M. (2011) Network-based functional enrichment, *BMC Bioinformatics*, **12 Suppl 13**, S14.

Rossin, E.J., *et al.* (2011) Proteins encoded in genomic regions associated with immune-mediated disease physically interact and suggest underlying biology, *PLoS Genet*, **7**, e1001273.

Scott, D.W. (1979) On optimal and data-based histograms, *Biometrika*, **66**, 605-610.

Sturges, H.A. (1926) The choice of a class interval, *Journal of American Statistical Association*, 65-66.
